# SHIP1 Deficiency in Inflammatory Bowel Disease Is Associated With Severe Crohn’s Disease and Peripheral T Cell Reduction

**DOI:** 10.3389/fimmu.2018.01100

**Published:** 2018-05-22

**Authors:** Sandra Fernandes, Neetu Srivastava, Raki Sudan, Frank A. Middleton, Amandeep K. Shergill, James C. Ryan, William G. Kerr

**Affiliations:** ^1^Department of Microbiology and Immunology, Upstate Medical University, Syracuse, NY, United States; ^2^Department of Neuroscience and Physiology, Upstate Medical University, Syracuse, NY, United States; ^3^Department of Biochemistry and Molecular Biology, Upstate Medical University, Syracuse, NY, United States; ^4^Department of Psychiatry and Behavioral Sciences, Upstate Medical University, Syracuse, NY, United States; ^5^Department of Medicine, University of California San Francisco, San Francisco, CA, United States; ^6^Division of Gastroenterology, Medicine, US Department of Veterans Affairs, San Francisco, CA, United States; ^7^Department of Chemistry, Syracuse University, Syracuse, NY, United States; ^8^Department of Pediatrics, SUNY Upstate Medical University, Syracuse, NY, United States

**Keywords:** SHIP1, Crohn’s disease, T cells, human inflammatory bowel disease, *INPP5D*, ATG16L1, fusion transcript

## Abstract

In our previous study, we observed a severe reduction in the Src homology 2-containing-inositol-phosphatase-1 (SHIP1) protein in a subpopulation of subjects from a small adult Crohn’s Disease (CD) cohort. This pilot study had been undertaken since we had previously demonstrated that engineered deficiency of SHIP1 in mice results in a spontaneous and severe CD-like ileitis. Here, we extend our analysis of SHIP1 expression in peripheral blood mononuclear cells in a second much larger adult Inflammatory Bowel Disease (IBD) cohort, comprised of both CD and Ulcerative Colitis patients, to assess contribution of SHIP1 to the pathogenesis of human IBD. SHIP1 protein and mRNA levels were evaluated from blood samples obtained from IBD subjects seen at UCSF/SFVA, and compared to healthy control samples. Western blot analyses revealed that ~15% of the IBD subjects are severely SHIP1-deficient, with less than 10% of normal SHIP1 protein present in PBMC. Further analyses by flow cytometry and sequencing were performed on secondary samples obtained from the same subjects. Pan-hematolymphoid SHIP1 deficiency was a stable phenotype and was not due to coding changes in the *INPP5D* gene. A very strong association between SHIP1 deficiency and the presence of a novel SHIP1:ATG16L1 fusion transcript was seen. Similar to SHIP1-deficient mice, SHIP1-deficient subjects had reduced numbers of circulating CD4^+^ T cell numbers. Finally, SHIP1-deficient subjects with CD had a history of severe disease requiring multiple surgeries. These studies reveal that the SHIP1 protein is crucial for normal T cell homeostasis in both humans and mice, and that it is also a potential therapeutic and/or diagnostic target in human IBD.

## Introduction

The global prevalence of inflammatory bowel disease (IBD) has been rising for the last several decades and is projected to continue doing so (1). While much progress has been made in understanding this heterogeneous disease, the causes of Crohn’s disease (CD) and ulcerative colitis (UC) are not fully known. Significant insights into the etiology of human IBD have come from a consortia of Genome-Wide Association Studies, which have identified more than 100 different inherited mutations that influence the risk of IBD in affected families (2). Nonetheless, only 8–12% of IBD cases in adults can be attributed directly to any of these risk alleles. Thus, the risk factors for approximately 90% of IBD patients have yet to be defined, and the exact cause for IBD remains unknown in most adult cases. Moreover, even in subjects with known mutations, the mechanisms by which they promote IBD are incompletely understood, and these genetic studies have not yet resulted in the development of new treatments for IBD.

Our current study derives from observation of the multiple roles of the SH2 domain-containing inositol polyphosphate 5′-phosphatase-1 (SHIP1) in immune regulation (3–6). SHIP1 antagonizes class I phosphatidylinositol 3-kinase signaling, which is critical in many cellular processes including immune activation. We initially showed that the engineered deficiency of SHIP1 in mice leads to ileitis with neutrophilia and a paucity of T cells in mice (7). Next, analysis of the SHIP1 protein level in PBMC from a small adult CD cohort (*n* = 35) at Erasmus MC, the Netherlands, indicated that a subpopulation of CD patients (*n* = 5) displayed profound SHIP1-deficiency (8). Here, using a large IBD patient cohort, we show that a significant subset of both CD and UC patients have dramatically low SHIP1 protein, and that SHIP1 deficiency is associated with a significant reduction in peripheral CD4^+^ T cell numbers in humans, as well as in mice. The gene encoding SHIP1, *INPP5D*, lies within 44 kb of ATG16L1, an autophagy gene implicated by Genome-Wide Association Studies in human IBD susceptibility (9). Despite this close physical proximity, SHIP1 deficiency is independent of *ATG16L1* risk alleles, and the SHIP1-deficient phenotype is not caused by germline-encoded mutations in *INPP5D*. Rather, PBMC from SHIP1-deficient subjects have a partially reduced expression of SHIP1 mRNA and express a novel SHIP1:ATG16L1 fusion mRNA splice variant. SHIP1 protein is subject to increased degradation by the ubiquitin-dependent proteasome system in SHIP1-deficient subjects. These reductions in SHIP1 expression appear to contribute to increased disease severity in CD patients.

## Materials and Methods

### Patient Recruitment

Subjects aged 21 and over with a firmly established diagnosis of either CD or UC were recruited from the IBD clinic at the SFVA Medical Center. All subjects signed written consent in accordance with the Institutional Review Boards at the University of California and the Veterans Affairs Medical Center, San Francisco. All studies were conducted in accordance with international human research guidelines. Some subjects provided additional blood samples upon request at a later date. Clinical information was obtained from examination of the electronic medical record and from direct patient interviews. The study was conducted in accordance with international research guidelines and was approved by the Institutional Review Boards at the University of California and Veterans Affairs Medical Center in San Francisco (Human Research Protection Program protocol 12-09140).

### Isolation of PBMC

Whole blood was subjected to density gradient centrifugation using Ficoll-Paque (GE Healthcare Life Sciences) according to the manufacturer’s recommendations. Leukocytes isolated from buffy coats were washed in PBS prior to resuspension in three pellet volumes of ice cold lysis buffer (20 mM Tris pH 7.5, 150 mM NaCl, 1 mM EDTA, 1 mM EGTA, and 1% Triton X-100) with 1× HALT Protease inhibitor (Pierce) and 1 mM phenylmethylsulfonyl fluoride (PMSF, Sigma). Lysates were incubated for 10 min on ice and then centrifuged at 21,000 × *g* at 4°C for 10 min. Cleared lysates were stored at −80°C.

### Quantitative Western Blot Analysis for SHIP1 Expression

Protein in each PBMC lysate was quantified using the BCA Protein Assay Kit (Pierce) according to manufacturer’s recommendation. Equal amounts of protein were separated on Mini-PROTEAN TGX 4–15% Precast gels (Bio-Rad Laboratories). The standard healthy control (HC) (C0) was obtained by mixing equal amounts of protein from each of the 24 HC samples. An aliquot of the C0 sample was run on each gel along with patient sample lysates and/or HC lysates. Proteins were transferred to nitrocellulose membranes using Turbo Trans-blot system (Bio-Rad Laboratories). The membranes were blocked with 5% non-fat dry milk in Tris Buffered Saline with 0.1% Tween-20 and probed with specific primary antibodies against SHIP1 (P1C1 or D11), actin (clone I-19), Src homology region 2 containing tyrosine phosphatase 1 (SHP1, SH-PTP1 clone C-19), heat shock protein 90 (Santa Cruz Biotechnology, Santa Cruz, CA, USA) or SHIP1 (D1163, Cell Signaling Technologies) and then by using the appropriate species-specific horseradish peroxidase conjugated secondary antibodies (Santa Cruz Biotechnology, Santa Cruz, CA, USA). Proteins were detected by using Super Signal ECL Substrate (Pierce). Band intensities were measured with ImageLab 4.0 software (Bio-Rad Laboratories), and the SHIP1 protein levels were normalized to beta-actin. The standard HC, C0, level was arbitrarily set as 100% and all other samples were quantified as relative to C0. SHIP1 values were recorded only if the housekeeping gene level varied by less than fivefold relative to C0 to ensure adequate sample loading in each well. When sample volumes permitted, samples were run in duplicate gels and relative SHIP1 levels were averaged between experiments.

### Quantitative Reverse Transcription-Polymerase Chain Reaction (qRT-PCR) Quantification of SHIP1 mRNA Expression

Quantitative reverse transcription-polymerase chain reaction was performed as previous described on RNA extracted from whole blood with primers listed in Table S1 in Supplementary Material (8). Briefly, 200 ng of RNA was used for cDNA synthesis and relative level of SHIP1 vs RPLP1 mRNA was compared between IBD subjects and HCs using the delta-delta Ct method. Data were cleaned to remove subjects with delta Ct values exceeding 2.55 SD from the average of the subjects group. A total of 1 control and 3 IBD samples were removed. Final sample number was 25 controls and 96 IBD samples.

### RT-PCR Analysis of SHIP1-ATG16L1 Fusion Transcript

cDNA were prepared as above. PCR was performed using the primers listed in Table S1 in Supplementary Material and positive and negative samples were evaluated on agarose gel. Primers for identification of the SHIP1:ATG16L1 transcript (Table S1 in Supplementary Material) were designed based on the sequence found though RNA-Sequencing (RNA-Seq) analysis.

### RNA-Sequencing

Whole transcriptome profiling was performed by using a stranded RNA-Seq approach by the SUNY Molecular Analysis Core at Upstate Medical University. Total RNA was purified by using aRNeasy kit (QIAGEN), followed by quantification and integrity assessment using the Agilent RNA 6000 Nano kit (Agilent Technologies). Libraries were generated using the TruSeq Stranded Total RNA Kit (Illumina). The samples were sequenced with the IlluminaNextSeq 500 instrument using 1 bp × 75 bp single end reads and a High Output V2 kit (Illumina), with a targeted average depth of coverage >30 million reads per sample. After obtaining the raw sequence reads, FASTQ files were uploaded into IlluminaBaseSpace for initial quality control, summarization, and alignment performed to hg19 of the human genome using the Bowtie2 algorithm within the TopHat application. The aligned.BAM file reads were downloaded into Strand next generation sequencing (NGS) (2.6) for gene fusion detection. Overall, we obtained a read depth of 32.1 million reads per sample, with 92.8% mapped to transcripts using the November 2015 Ref-Seq gene annotation model. An average of 99.4% of all reads was stranded, and approximately 75% of the reads were located fully within an exon, with the remainder non-exonic or ambiguous. When summarized by gene, there were a total of 42,497 unique genes identified in the data, encompassing 147,158 different transcripts. The present report solely uses RNA-Seq expression data on the *SHIP1* and *ATG16L1* transcripts.

### *INPP5D*Exome Sequencing

Genomic DNA was extracted from whole blood using the QIAamp DNA Blood Mini Kit (QIAGEN). Thirty individual PCR reactions were performed using primers designed to amplify the 27 exons from the *INPP5D* gene (University of California Santa Cruz Genome Browser, GRCh38/hg38) on 8 SHIP1-deficient, 8 SHIP1-sufficient, and 8 HC samples with Phusion High-Fidelity DNA Polymerase in HF buffer (New England Biolabs). Each amplicon was submitted for sequencing at McLabs, Inc. (San Francisco, CA, USA) and sequences were compared to the Gencode transcripts ENST00000445964.4. Primers used for amplification are listed in Table S2 in Supplementary Material.

### *ATG16L1* SNP Genotyping

Ten nanograms of genomic DNA (extracted as above) were placed in triplicate well in 384-well plates and left to dry for several hours at room temperature in an amplicon-free laminar flow hood. TaqMan SNP Genotyping Assay rs2241880 (ThermoFisher Scientific) was performed according to manufacturer’s recommendations with TaqMan Genotyping Master Mix (ThermoFisher Scientific) on the LightCycler 480 (Roche). Endpoint Genotyping Analysis was performed on each sample using the LightCycler 480 software (Roche).

### Proteasome Inhibition and Flow Cytometry Analysis of Human Blood Samples

Whole blood was diluted 10-fold in 1× RBC lysis buffer (ThermoFisher Scientific) for 10 min on ice, followed by 10 min centrifugation at 350 × *g* at 4°C. Supernatants were decanted and cell pellets were resuspended in 1 ml of culture media [RPMI (ATCC), 10% fetal bovine serum (Sigma)] and distributed into 24-well plates. Samples were then treated with vehicle [dimethyl sulfoxide (DMSO) (Sigma) 0.1% final in culture media], Mg132 [10 µM final in DMSO (Sigma)], or PR-619 [DUBi, 50 µM final in DMSO (Sigma)] for 1 h at 37°C under 5% CO_2_ atmosphere. After 1 h, cells were washed twice with 2 ml cold PBS and centrifuged for 5 min at 350 × *g* at 4°C. Dead cells were stained with Live/Dead Fixable Aqua Dead Cell Stain (ThermoFisher Scientific) according to manufacturer’s recommendations, washed, and resuspended in staining media (3% FBS, 10 mMHepes pH 7.0 in PBS, 50% BD Brilliant stain buffer) with the following surface staining anti-human antibody cocktail: [BB515-conjucated anti-CD3 BB515, BV786-conjucated anti-CD15 (BD Biosciences)], [PerCP-Cy5.5-conjucated anti-CD4, PE-Cy7-conjucated anti-CD56, APC-conjucated anti-Siglec8, AlexaFluor700-conjucated anti-CD16, APC-Cy7-conjucated anti-CD8a, BV605-conjucated anti-CD19, and BV711-conjucated anti-CD14 (BioLegend)]. Following incubation, cells were washed with PBS, centrifuged for 5 min at 350 × *g*, decanted, and resuspended in IC Fixation Buffer (ThermoFisher Scientific) for 25 min. Cells were washed with permeabilization buffer (ThermoFisher Scientific), Fc receptors were blocked with human Fc receptor binding inhibitor (ThermoFisher Scientific) and stained with PE-conjugated anti-SHIP1 (BioLegend). Samples were washed with permeabilization buffer, centrifuged 5 min at 400 × *g* and resuspended in permeabilization buffer. All samples were acquired on LSR-Fortessa (BD Biosciences) immediately after staining, and data were analyzed using FlowJo software.

### CD4CreSHIP^flox/flox^ and SHIP^flox/flox^ Mice

CD4Cre mice transgenic mice were initially purchased from Jackson Laboratories (Bar Harbor, ME, USA), were bred to SHIP^flox/flox^ mice (3) and then maintained in a pathogen-free environment at Upstate Medical University as previously described (10). All animal experiments were approved by the Upstate Medical University Institutional Animal Care and Use Committee.

### Flow Cytometry on Mouse Blood

Red blood cells were lysed from whole mouse blood with 1× RBC Lysis Buffer (ThermoFisher Scientific) and cells were stained with the following anti-mouse antibody cocktail [PE-Cy7-conjucated anti-CD3, PerCP-Cy5.5-conjucated anti-NK1.1, FITC-conjucated anti-CD4 (ThermoFisher Scientific), and APC-Cy7-conjucated anti-CD8 (BD Biosciences)] for 30 min. Following washes, cells were resuspended in DAPI dye for dead cell exclusion prior to acquisition on LSR-Fortessa (BD Biosciences) as described above.

### Statistical Analysis

All statistical analyses were performed using the Prism 5.0 statistical software (GraphPad, San Diego, CA, USA).

## Results

### A Significant Subset of Both CD and UC Is Severely SHIP1-Deficient at the Protein Level

**Figure 1:**
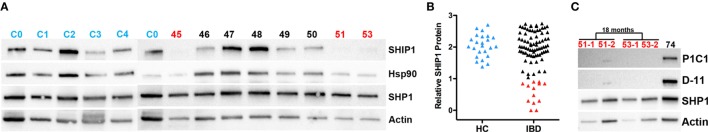
SHIP1 protein for a subset of the 89 samples quantified from the University of California San Francisco/San Francisco Veteran’s Affairs cohort. **(A)** Western blots for SHIP1 (P1C1, Santa Cruz), β-actin, SHP1, and heat shock protein 90 (Hsp90) on a subset of inflammatory bowel disease (IBD) patient lysates (45–53, in black for SHIP1-sufficient and red for SHIP1-deficient samples as determined in B), HC samples (C1–C3, in blue) and the standard control C0. The C0 sample, pooled from equal amount of protein from all 24 HCs samples, was run with each Western blot to quantify relative SHIP1 expression levels in each sample. **(B)** Log10 of quantitative Western blot values of SHIP1 normalized to beta-actin as compared to C0 (arbitrarily set to 100% = 10^2^). HC samples are shown in blue, and the SHIP1 deficiency threshold was set at 10% (10^1^). SHIP1-sufficient IBD samples are in black (*n* = 76) and SHIP1-deficient samples, displaying less than 10% of SHIP1 in C0, are shown in red (*n* = 13). **(C)** Lysates prepared from PBMC from SHIP1-deficient patients (51-2 and 53-2) taken 18 months after the initial blood draw was probed in parallel to an aliquot of the initial samples (51-1, 53-1) and a SHIP1-sufficient sample. This has been confirmed in several SHIP1-deficient patients. Western blots were also re-probed with different antibodies against SHIP1 (D1163, Cell Signaling Technologies, not shown and D-11, Santa Cruz) that recognize distinct epitopes on the protein.

SHIP1 protein expression was analyzed in nearly 100 adult IBD subjects with CD or UC. After normalization of the SHIP1 levels to the housekeeping gene beta-actin, we defined a threshold for SHIP1 deficiency to be less than 10% of the SHIP1 protein level found in the pooled HX (C0) sample (Figure 1A). Using this value, 14.6% (13 out of 89) of the adult IBD population in our cohort demonstrate a profound degree of SHIP1 protein deficiency (<10% of normal, range 0.5–8.0%, Mean ± SD, 4.5 ± 2.8%) in PBMC (Figure 1B). To confirm that the lack of detection of SHIP1 protein was not simply due to allelic variation leading to a loss of antibody binding, the samples were re-probed using an antibody that recognizes a different epitope in the SHIP1 protein (Figure 1C). Importantly, the SHIP1-deficient phenotype appeared to be stable over time, as lysates from sequential blood draws taken several months apart showed consistently low SHIP1 in reanalyzed SHIP1-deficient subjects. Importantly SHIP1 deficiency was identified in some subjects in complete remission without IBD medications (e.g., see BK051 and BK053, Figure 1C).

### SHIP1 Deficiency Is Associated With Severe Complications of CD

**Figure 2:**
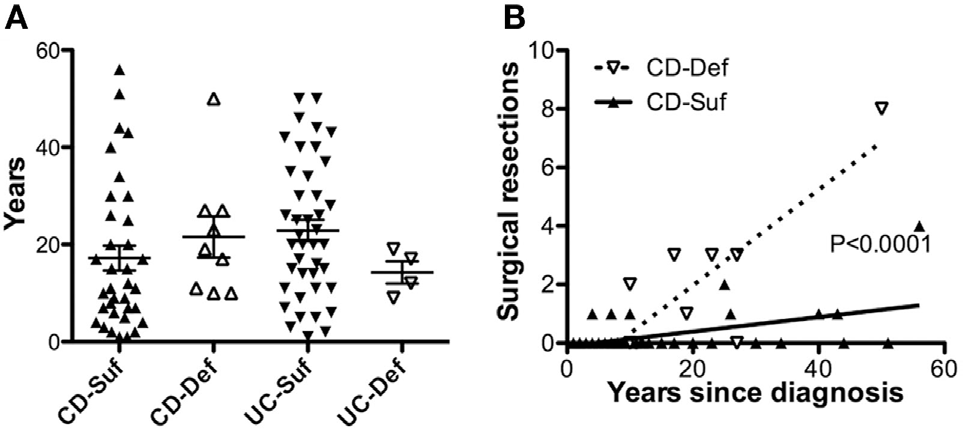
SHIP1-deficient Crohn’s disease (CD) subjects have a history of more severe disease that does not result from a longer duration of disease. **(A)** No differences were observed in the duration of disease at the time of enrollment between SHIP1-sufficient and SHIP1-deficient subjects with either disease subset. **(B)** Increased number of surgical resections is not due to a longer duration of disease in SHIP1-deficient CD patients (Linear regression analysis, *P* < 0.0001).

Nearly 100 IBD adult patients were enrolled in the IBD cohort during this study and their clinical characteristics are presented in Table 1. The cohort was 97% male as it was recruited in a VA medical center, with an average age of 60.7. A total of 13 patients were SHIP1-deficient, including 9 with CD and 4 with UC. No significant differences were observed in the age at diagnosis (not shown) or in disease duration between SHIP1-deficient and SHIP1-sufficient subjects regardless of their type of disease (Figure 2A). A history of IBD in first or second-degree relatives was significantly elicited in SHIP1-deficient 6/13 (42.9%) as compared to SHIP1-sufficient subjects 12/85 (14.1%) in the cohort (*P* = 0.0154) (not shown). Comparing each IBD disease subset individually, family history of disease also reached significance in subjects with UC (3/4, *P* = 0.0296). A history of severe disease requiring surgical resection was significantly higher in SHIP1-deficient CD subjects (*P* = 0.0194). In addition, a significantly higher percentage of SHIP1-deficient CD subjects required two or more surgical resections as compared to SHIP1-sufficient CD patients (5/9 vs 2/35, *P* = 0.0022). Linear regression analysis indicated that this increased requirement for resections was not simply the result of longer disease history in SHIP1-deficient vs SHIP1-sufficient CD subjects (Figure 2B). Finally, there was also distinct trend toward increased intestinal neoplasia (dysplasia or cancer) and toward an increased use of aggressive treatments (anti-TNFα or anti-α_4_β_7_ integrin) in SHIP1-deficient CD. Together, these data suggest that SHIP1 deficiency in CD subjects is likely associated with a more aggressive disease course.

**Table 1:** Clinical characteristics of the IBD subjects in the cohort.

	Crohn’s disease			Ulcerative colitis		
		
	SHIP1-Def	SHIP1-Suf	*P*-value	SHIP1-Def	SHIP1-Suf	*P*-value
Number of subjects	9	35		4	41	
Mean age	56.2	58.3		60.0	63.4	
Mean age at diagnosis	34.7	41.6	0.2251	45.8	40.5	0.5400
Mean duration of disease	21.6	17.5	0.4330	14.3	22.9	0.2337
Family history	3 (33.3%)	4 (11.4%)	0.1383	3 (75.0%)	7 (17.1%)	0.0296
With resections	6 (66.7%)	8 (22.9%)	0.0194	0 (0.0%)	9 (22.0%)	0.5687
2+ resections	5 (55.6%)	2 (5.7%)	0.0022	0 (0.0%)	0 (0.0%)	N/A
IBD-related neoplasia	2 (22.2%)	1 (2.9%)	0.1015	0 (0.0%)	5 (12.2%)	1
Use of biologics	6 (66.7%)	14 (40.0%)	0.2607	1 (25.0%)	5 (12.2%)	0.4480

### No Coding Mutations Were Found in the *INPP5D* Gene

Exome sequencing of the entire *INPP5D* gene including the promoter region on eight SHIP1-deficient and eight SHIP1-sufficient IBD subjects as well as from eight HCs failed to reveal any disruptive coding changes in the SHIP1-deficient subjects (data not shown). Furthermore, RNA-Seq (RNA-Seq) analysis of the whole blood transcriptome of eight SHIP1-deficient patients confirmed the absence of point mutations in the *INPP5D* open-reading frame (data not shown).

### SHIP1-Deficient Subjects Express SHIP1 mRNA

**Figure 3:**
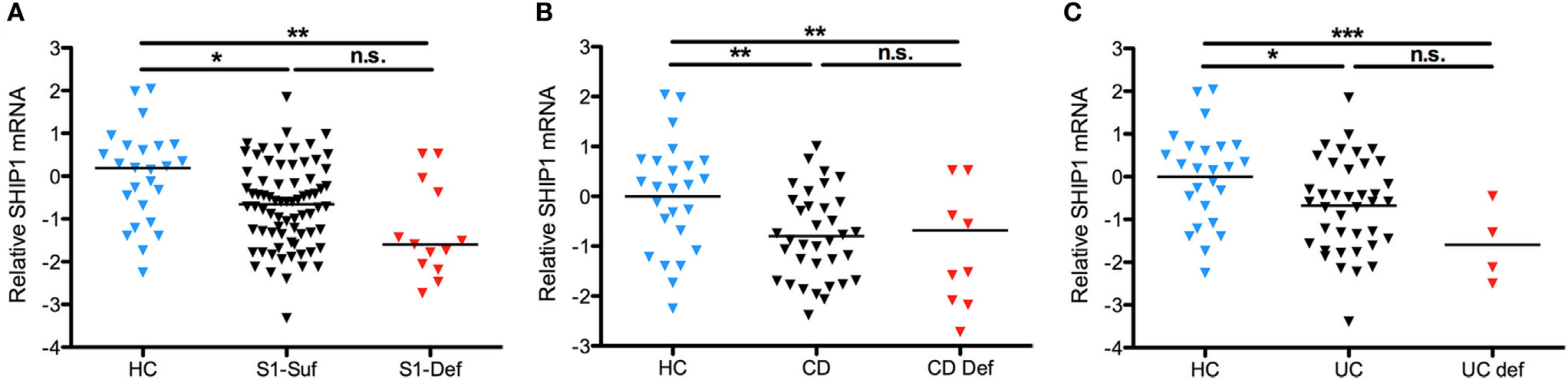
SHIP1 mRNA is reduced in both CD and ulcerative colitis (UC) as compared to healthy controls. **(A)** Log2 of SHIP1 mRNA levels relative to RPLP1 between healthy control (HC, *n* = 25) and either SHIP1-sufficient (*n* = 74) or SHIP-deficient (*n* = 13) IBD subjects. **(B,C)** Log2 Relative SHIP1 mRNA levels were not significantly different between SHIP1-Sufficient CD (CD Suf *n* = 34) or SHIP1-Deficient CD (CD Def, *n* = 9) subjects **(B)** nor between SHIP1-Sufficient UC (UC Suf *n* = 40) or SHIP1-Deficient UC subjects (UC Def, *n* = 4) **(C)** although they were all significantly reduced as compared to healthy controls (two-tailed *t*-test with Welch’s correction for unequal variances, * *P* < 0.05, **P < 0.01, ***P < 0.001).

To determine if SHIP1 deficiency was triggered by reduced transcription of *INPP5D*, we analyzed the level of SHIP1 mRNA in leukocytes by qRT-PCR (vs the housekeeping gene *RPLP1*) using probes located at the 5′ end of the SHIP1 mRNA transcript. Total SHIP1 mRNA levels were decreased in both SHIP1-sufficient and SHIP1-deficient IBD subjects as compared to healthy controls suggesting that reduced transcription is not responsible for reduced SHIP1 protein expression in SHIP1 deficient patients (Figure 3A), and this was also seen for each individual disease subset (Figure 3B for CD and Figure 3C for UC).

### A Novel mRNA Fusion Transcript Links Exon 25 of SHIP1 to Exon 2 of ATG16L1 in Most SHIP1-Deficient Subjects

**Figure 4:**
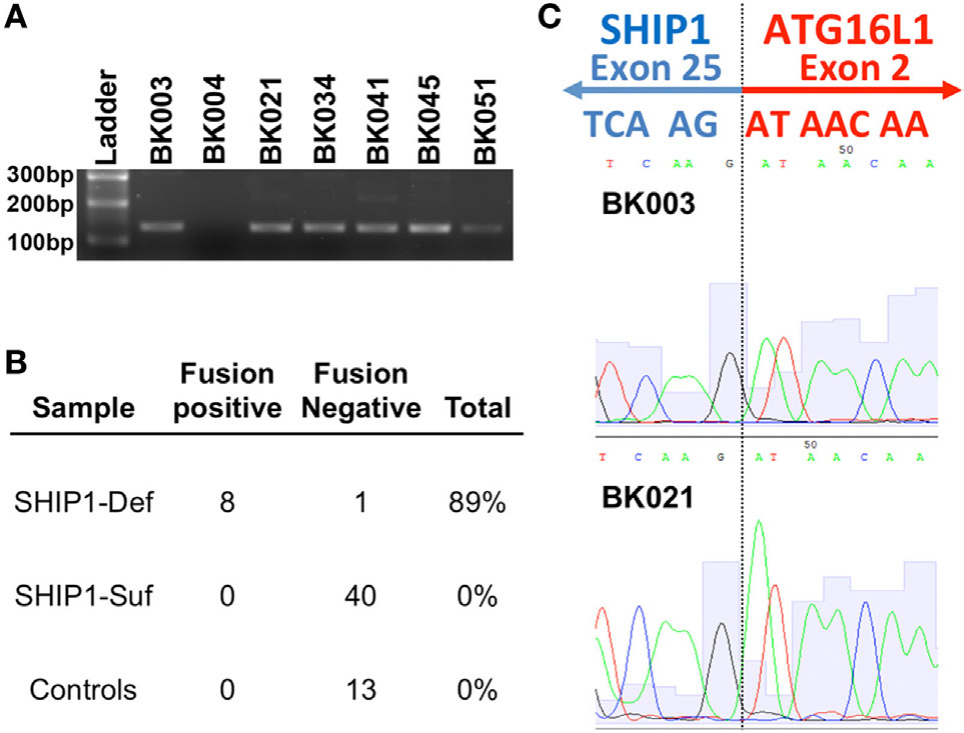
RNA-sequencing (RNA-Seq) analysis uncovered a novel fusion mRNA transcript that links SHIP1 exon 25 to ATG16L1 exon 2 in SHIP1-deficient subjects. **(A)** RNA-Seq analysis revealed the presence of a fusion transcript between SHIP1 and downstream ATG16L1 in 8 of 8 SHIP1-deficient subjects but not in either 2/2 of the SHIP1-sufficient patient analyzed in the same analysis. RT-PCR analysis using primers specifically targeting the fusion transcript was used to screen a subset of the inflammatory bowel disease (IBD) cohort, which confirmed the presence of this fusion transcript in 8/9 SHIP1-deficient patients. **(B)** Fusion transcript was not observed in 53 of the SHIP1-sufficient and healthy control subjects, while it was observed in 89% of SHIP1-deficient samples (8 of 9). This represents a highly significant association to the SHIP1-deficient phenotype with an odds ratio (OR) of 153.0 (95% CI: 5.562–4,209, *P* < 0.0001) compared to the 13 controls, and an OR of 606.3 (95% CI: 22.76–16,150, *P* < 0.0001) compared to all available SHIP1-sufficient IBD and control subjects (*n* = 53). **(C)** Junction of exon 25 of SHIP1 mRNA to exon 2 of ATG16L1 as identified by RNA-Seq was confirmed by sequencing of the fusion RT-PCR product.

RNA-sequencing analysis from SHIP1-deficient patients revealed the presence of a novel fusion transcript linking the SHIP1 transcript to downstream ATG16L1 transcripts in all eight SHIP1-deficient patients examined (Figure 4A). This fusion transcript was confirmed and identified by subsequent RT-PCR analysis in eight of nine SHIP1-deficient subjects analyzed, and no fusion transcript could be identified in any of the 40 SHIP1-sufficient IBD subjects or in the 13 healthy control subjects analyzed (Figure 4B). Overall, the presence of this fusion transcript represents a highly significant novel association with the SHIP1-deficient phenotype, with a conservatively estimated odds ratio (OR) of 153.0 (95% CI: 5.562–4209, *P* < 0.0001) compared to the healthy 13 controls, and an OR of 606.3 (95% CI: 22.76–16,150, *P* < 0.0001) compared to all available SHIP1-sufficient IBD and healthy control subjects (*n* = 53).

While the molecular mechanism leading to the generation of the SHIP1:ATG16L1 fusion transcript is currently under investigation, analysis of the sequence reads indicates that it would link exon 25 of *INPP5D* to exon 2 of *ATG16L1* where it would create a stop codon (Figure 4C). Translation of this fusion transcript would thus result in generation of a truncated SHIP1 protein (991 amino acids) with an apparent molecular weight of 112 kDa. The predicted sequence of this 112 kDa protein includes the SHIP1 catalytic domain and the N-terminal SH2-domain but omits both NPXY domains and the Proline-rich region that interacts with SH3-domain-containing proteins that are encoded by exons 26 and 27. Since no C-terminally truncated protein of this size was observed on Western blots using several different anti-SHIP1 antibodies (data not shown), this carboxy terminal truncation of SHIP1 may be unstable in PBMC and hence rapidly degraded. Moreover, whole-genome NGS analysis performed on two SHIP1-deficient fusion positive samples failed to uncover any obvious genetic signature (e.g., a genomic deletion or inversion) that would account for generation of this novel transcript (data not shown). Thus, the presence of the fusion appears to be a relatively stable epigenomic feature of SHIP1-deficiency.

### SHIP1 Deficiency Does Not Correlate to *ATG16L1* rs2241880 Risk Allele

Homozygosity for the rs2241880 IBD risk allele (GG) within the autophagy complex protein ATG16L1 has been previously correlated to low, but easily detectable, SHIP1 mRNA in a small pediatric CD cohort (11). This association was seen in both CD and healthy control subjects. The ATG16L1 risk allele (G) encodes a single non-synonymous adenine to guanine transition that leads to a threonine to alanine substitution at position 300 (T300A) of ATG16L1. The T300A substitution renders the ATG16L1 protein susceptible to caspase cleavage, potentially leading to altered stoichiometry of the autophagosome and to defective autophagy (12). In our cohort, we found no correlation between the rs2241880 genotype and SHIP1 mRNA (Figure S1A in Supplementary Material) or between SHIP1 protein levels in IBD (Figure S1B in Supplementary Material) or in CD subjects (Figure S1C in Supplementary Material).

### Profound SHIP1 Protein Reductions Are Present in All Major Lymphoid and Myeloid Lineages in SHIP1-Deficient IBD Patients

**Figure 5:**
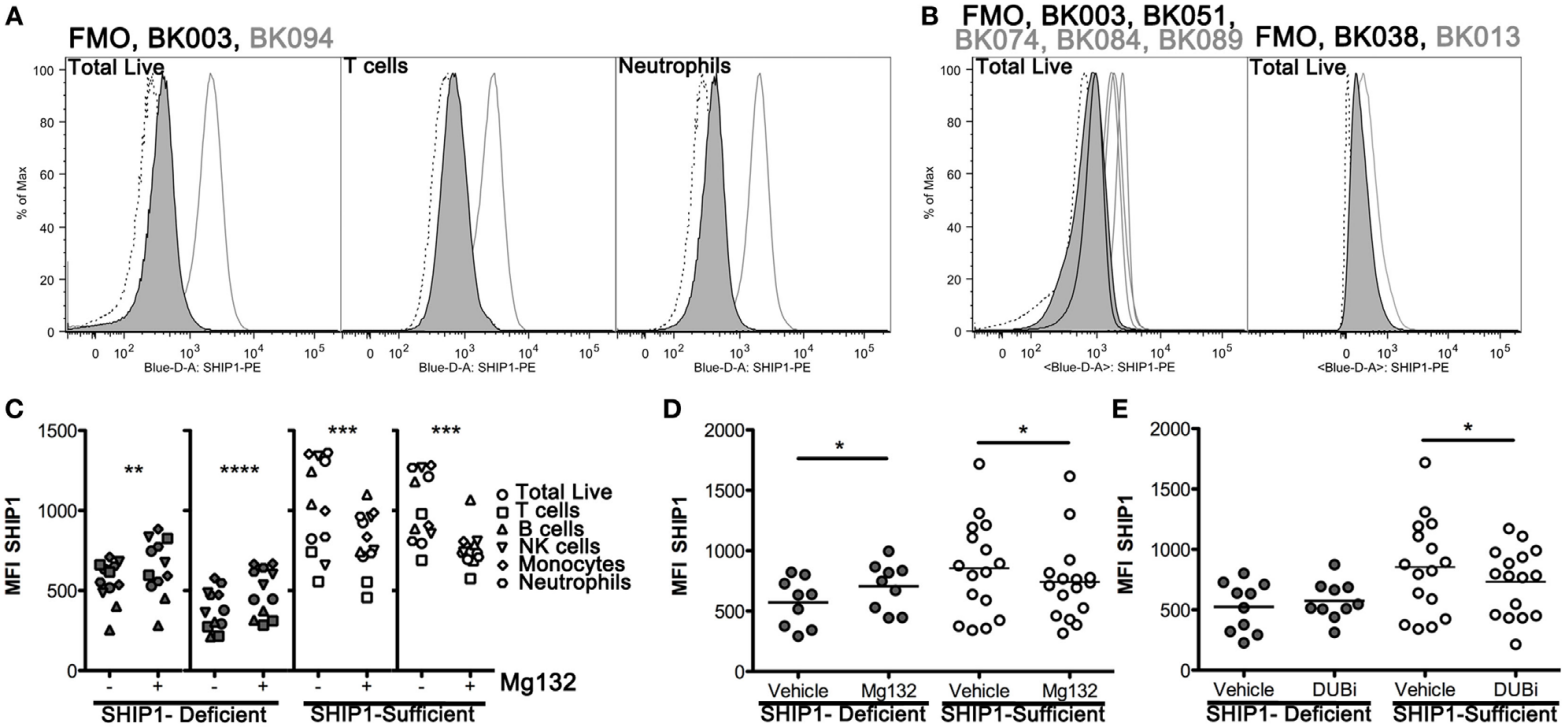
SHIP1 deficiency is observed in all major blood lineages and is subject to increased proteasome-mediated degradation in SHIP1-deficient inflammatory bowel disease (IBD) patients. **(A)** Histograms for SHIP1 staining by intracellular flow cytometry (icFlow) performed on leukocytes from a SHIP1-deficient (BK003, black line-gray fill) and SHIP1-sufficient (BK094, gray line) IBD patient and fluorescence-minus-one (FMO, dotted line). Samples were gated for total live cells, T cells (CD3^+^), and neutrophils (CD15^+^ CD16^+^). **(B)** Histograms for total live cells for several SHIP1-deficient (black line-gray fill) and SHIP1-sufficient (gray line) patients, from independent experiments along with FMO (dotted line) for each sample. **(C)** Leukocytes from two individual SHIP1-deficient (gray) and 2 SHIP1-sufficient (white) subjects were cultured *ex vivo* for 1 h in the presence of Mg132 (+) or Vehicle alone (−, 0.1% dimethyl sulfoxide final) and analyzed for SHIP1 expression in major blood cell types by icFlow. **(D)** Effect of Mg132 treatment on total live cells from several subjects. **(E)** Treatment of cells with pan-deubiquitinase inhibitor PR-619 (DUBi) has opposite effects on total live cells from SHIP1-deficient and SHIP1-sufficients subjects. [For **(D,E)**, Filled circles: SHIP1-deficient *n* = 6, Open circles: SHIP1-sufficient *n* = 9. Two-tailed, paired t-tests, *P < 0.05, **P < 0.01, ***P < 0.001, ****P < 0.0001.]

To examine potential mechanisms for altered SHIP1 protein stability, we developed an intracellular flow cytometry (icFlow) assay to analyze SHIP1 protein levels in primary blood cells, including CD4^+^ and CD8^+^ T cell, B cells, NK cells, monocytes, and neutrophils. SHIP1 deficiency was detected across all major cell lineages in the SHIP1-deficient subject BK003, but not in SHIP1-sufficient subject BK094 (Figure 5A; Figure S2 in Supplementary Material). This pan-hematolymphoid downregulation of SHIP1 was confirmed in other SHIP1-deficient subjects (BK051, BK038, and in a second sample from BK003, Figure 5B).

### SHIP1 Protein Is Increased by Blockade of the Proteasome in SHIP1-Deficient but Not in SHIP1-Sufficient Subjects

Steady state expression of SHIP1 protein can be reduced by the ubiquitin-proteasome degradation system (13, 14). We, therefore, considered the possibility that the reduction of SHIP1 in deficient subjects may be due to selective or aberrant protein degradation. For this purpose, we treated leukocytes with the proteasome inhibitor Mg132 for 1 h before staining the cells for icFlow. This brief *ex vivo* treatment allowed for partial rescue of SHIP1 protein levels across all major blood cell types, including T cells, in SHIP1-deficient subjects (Figure 5C, two left panels), but not in SHIP1-sufficient subjects (Figure 5C, right two panels). When assayed across several subjects in our cohort, Mg132 treatment induced a significant increase in SHIP1 MFI in the deficient subjects, while it has the opposite trend in SHIP1-sufficient subjects, significantly decreasing SHIP1 expression in all lineages (live cells shown, Figure 5D). The preferential degradation of SHIP1 in deficient samples might be due to a loss of expression or a loss of function mutation in a deubiquitinase (DUB) that selectively targets SHIP1 to prevent its inappropriate degradation. This hypothesis is supported by the observation that treatment of leukocytes from SHIP1-deficient patients with DUB inhibitor PR-619 resulted in no loss of SHIP1, while the same treatment on leukocytes from SHIP1-sufficient subjects had the opposite effect (live cells shown, Figure 5E). Taken together, these results suggest that SHIP1 deficiency might result, in part, from alterations in the expression or function of regulatory gene(s) involved in the ubiquitin-proteasome system.

### SHIP1-Deficient IBD Subjects and SHIP1 Conditional Mutant Mice Exhibit Profound Reductions in Circulating CD4^+^ T Cell Numbers

**Figure 6:**
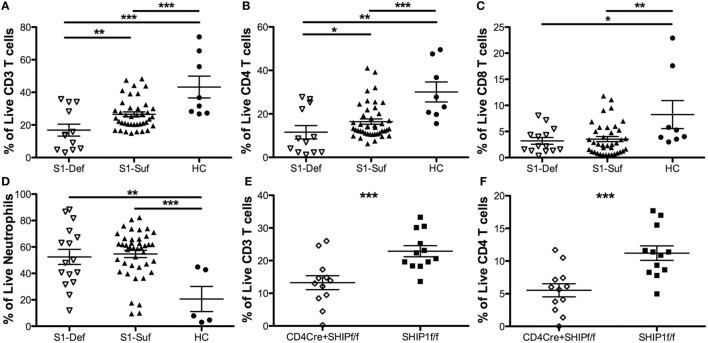
SHIP1-deficient inflammatory bowel disease (IBD) patients have significantly reduced CD4 T cell numbers relative to other IBD patients and healthy controls, similar to mice with a conditional mutation of SHIP1 in T lymphocytes. Frequency of CD3^+^ T cells **(A)**, CD4^+^ T cells **(B)**, CD8 T cells **(C)**, and neutrophils **(D)** were compared among SHIP1-deficient (S1-Def), SHIP1-sufficient (S1-Suf), and healthy control (HC). Leukocytes cultured *ex vivo* for 1 h in complete media prior to intracellular flow cytometry staining for SHIP1. (Data points were pooled from multiple independent analyses, one-tailed *t*-test, *P < 0.05, ***P < 0.001, ****P < 0.0001. Duplicate samples were analyzed for each subject, total number of independent subjects analyzed: S1-Def = 6, S1-Suf = 21, HC = 4.) **(E)** Frequency of live and CD3^+^ T cells **(E)** and CD4^+^ T cells **(F)** in leukocytes of CD4CreSHIP^flox/flox^ and SHIP^flox/flox^ control mice (one-tailed *t*-test ***P < 0.001, *n* = 12/group).

We sought to determine the impact that SHIP1 protein loss might have on the homeostasis of circulating blood cells. We observed a significant decrease in total CD3^+^ T cell numbers in the blood of SHIP1-deficient compared with SHIP1-sufficient IBD patients and healthy controls (Figure 6A). This deficit was primarily due to reduced CD4^+^ T cell numbers (Figure 6B) although CD8^+^ T cell frequencies were also reduced in both IBD patient subsets as compared to controls (Figure 6C). Neutrophilia, as defined by an increased frequency of CD15^+^ CD16^+^ cells was also observed in both SHIP1-deficient and SHIP1-sufficient IBD subjects compared to healthy controls (Figure 6D). In mice, SHIP1 deficiency in both the T cell and myeloid lineages leads to increased ileitis relative to lineage knockouts of SHIP1 in either T cells or myeloid cells alone (15). Mice with a T cell lineage knockout of SHIP1 also have reduced circulating CD3^+^ CD4^+^ T cells (Figures 6E,F) and reduced CD3^+^ CD8^+^ T cells (data not shown), demonstrating that SHIP1 is intrinsically required for the maturation or survival of circulating T cells (Figures 6E,F). Since SHIP1-deficient humans also have low CD4^+^ T cells numbers, an intrinsic role for SHIP1 in promoting normal T cell homeostasis may be conserved across species. These findings suggest that a reduction in T cell numbers combined with an increase in neutrophils might potentially contribute to increased disease severity in SHIP1-deficient subjects.

## Discussion

Prompted by studies of SHIP1-deficient mice (7) and by our pilot study using a small CD patient cohort (8), we show here that SHIP1 protein is markedly reduced in a significant subset both CD and UC patients. We also found a statistically significant association of SHIP1 deficiency with severe complications of CD. As with SHIP1 deficiency in mice, SHIP1-deficient IBD patients exhibited a profound reduction in circulating CD4^+^ T cell numbers. In a previous study, we showed that human T cells experience significant Fas/Caspase eight mediated extrinsic cell death when treated with the SHIP1 selective inhibitor 3AC. Moreover, we showed that SHIP1 is recruited to the CD95/Fas receptor in human T cells. We validated that these *in vitro* findings in human T cells are germane to the T cell demise observed in the gut of SHIP1^−/−^ mice as a Caspase 8 inhibitor treatment of SHIP1^−/−^ mice rescued CD3^+^ T cell numbers in the gut and reduced intestinal inflammation. Thus, these combined *in vivo* studies in mice and *in vitro* studies with a human T cell line have previously provided a mechanistic underpinning for the current studies which now show that CD4 T cells numbers are also reduced in SHIP1-deficient patients (10). Thus, perturbations in the Fas-Caspase 8 pathway due to SHIP1 deficiency might account for the decline in mature T cells in these patients. The role of altered T cell homeostasis in the pathogenesis of IBD remains unclear. In SHIP1-deficient mice, regulatory T cells (Tregs) were slightly increased suggesting that inflammation in SHIP1 deficiency is not due to impaired Treg homeostasis (10).

The mechanism underlying SHIP1 deficiency in adult IBD subjects is not clear. *In vitro* proteasome inhibition led to significant increases in SHIP1 protein amounts in leukocytes from SHIP1-deficient subjects, but not in SHIP1-sufficient patients. These findings point to a preferential degradation of SHIP1 protein in a subset of IBD patients. Reduced SHIP1 protein expression did not result from coding changes in *INPP5D* exons or from transcriptional silencing as SHIP1 mRNA was detectable in both SHIP1-deficient and -sufficient IBD subjects. As observed in our pilot study (8), no correlation was found between SHIP1-deficiency and presence of the rs2241880 risk allele within the adjacent *ATG16L1* gene.

Interestingly, RNA-Seq analyses detected a novel SHIP1:ATG16L fusion mRNA transcript in nearly all (8/9) SHIP1-deficient IBD subjects analyzed, but in none of the SHIP1-sufficient IBD subjects or healthy controls examined. Although SHIP1 mRNA was detectable in all patients with the fusion, SHIP1:ATG16L1 fusion transcription would lead to reduced levels of full-length SHIP1 transcripts. The fusion transcript is unlikely to produce significant quantities of biologically active SHIP1, as the truncated SHIP1 protein appears to be unstable. Nonetheless, it is possible that increased degradation of truncated SHIP1 protein might also contribute to altered stability or dysregulation of the entire SHIP1 protein pool, leading to the deficiency of wild-type SHIP1 in affected subjects. The underlying mechanism behind the generation of this fusion mRNA remains unclear at present. We performed whole-genome sequencing of two SHIP1-deficient subjects, but these genomic data failed to highlight a genetic signature that would result in generation of this novel transcript (not shown). While short-read Illumina sequencing can detect most important genomic signatures, however, it can miss subtle or cryptic genomic features such as inversions, duplications, or trans-splicing signatures (16–20). It will, therefore, require additional exhaustive genomic analyses before we can firmly rule out a cryptic genomic signature as a cause of this novel fusion transcript.

Taken together, our findings also suggest that SHIP1 screening of both pediatric and adult IBD patients may identify those with impending severe disease that will ultimately require surgical resections. Although it did not reach statistical significance in the current cohort, more extensive analysis might also reveal an increase in IBD-associated neoplasia in SHIP1-deficient CD patients relative to other CD patients. Thus, assessments of SHIP1 expression in patients soon after diagnosis could identify CD patients who need more aggressive (“top down” rather than “step up”) treatments in order to prevent future disease complications.

Further analyses of the molecular basis of SHIP1 deficiency might also reveal potential molecular targets enabling the rescue of SHIP1 protein expression in IBD. Pharmacologic proteasome inhibition using Mg132 reduces intestinal inflammation by decreasing intestinal TNFα mRNA in IL10-deficient mice with a CD-like illness (21) and bortezomib, a clinically approved proteasome blocker, attenuates intestinal inflammation in animal models of colitis induced by dextran sulfate (22). Comparative transcriptional and functional profiling of ubiquitin ligases or deubiquinating enzymes in SHIP1-deficient and -sufficient patients may also reveal novel targets for pharmacologic inhibitors or agonists that can be used to restore or augment SHIP1 protein function in deficient patients.

## Ethics Statement

The study was conducted in accordance with international research guidelines and was approved by the Institutional Review Boards at the University of California and Veterans Affairs Medical Center in San Francisco (Human Research Protection Program protocol 12-09140).

## Author Contributions

SF, NS, and RS processed samples and collected data, SF, NS, and FM analyzed the sequencing data, AS and JR coordinated with the patients for collection of samples, SF, JR, and WK designed the study, analyzed data, and wrote the manuscript.

## Conflict of Interest Statement

SF, FM, and WK have a patent pending concerning the use of the SHIP1 nucleic acid detection techniques to detect and predict disease prognosis in IBD. All other authors have no conflicts to disclose.

## Abbreviations

DMSO, dimethyl sulfoxide; DUB, deubiquitinase; icFlow, intracellular flow cytometry; OR, odds ratio; SHIP1, Src homology 2-containing-inositol-phosphatase-1; SHP1, Src homology region 2 containing tyrosine phosphatase 1; Tregs, regulatory T cells; UCSF/SFVA, University of California San Francisco/San Francisco Veteran’s Affairs.
