# Supplementary material for: SHIP1 Deficiency in Inflammatory Bowel Disease Is Associated With Severe Crohn’s Disease and Peripheral T Cell Reduction
Source: Front Immunol. 2018 May 22;9:1100. doi: 10.3389/fimmu.2018.01100 (PMC5972310; doi:10.3389/fimmu.2018.01100)
Supplement: Supplementary file 1 [file data_sheet_1.DOCX]

Supplementary Material

SHIP1 Deficiency in Inflammatory Bowel Disease Is Associated with Severe Crohn’s Disease and Peripheral T Cell Reduction

Sandra Fernandes, Neetu Srivastava, Raki Sudan, Frank A. Middleton, Amandeep K. Shergill, James C. Ryan, William G. Kerr^*^

*** Correspondence:** William G. Kerr, kerrw@upstate.edu

# Supplementary Material and Methods

## Supplementary Tables

| **Supplementary Table 1. Primer sequences (5’-3’) used for RT-PCR assays** | | |
| --- | --- | --- |
| **Gene** | **Forward Primer** | **Reverse Primer** |
| SHIP1-A | GCGTGCTGTATCGGAATTGC | TGGTGAAGAACCTCATGGAGAC |
| SHIP1-B | AAGTGTCGTGTCTCCACCC | CGGGGATTCTCGTTTGAAAAAGG |
| RPLP1 | AGCCTCATCTGCAATGTAGGG | TCAGACTCCTCGGATTCTTCTTT |
| Fusion | CCCATATCACCCAAGAAGTT | AAATACCTTATCTCGTGCCTG |

| **Supplementary Table 2. Primers used to amplify the 27 exons of the *INPP5D* gene for exome-sequencing** | | |
| --- | --- | --- |
|  | **Forward Primer** | **Reverse Primer** |
| PromoterA | GGTTCCCACAGCTTGAATGT | TGTGACCCCATTACCCTCAT |
| PromoterB | TGTGTCTCCAAGCTGGACTG | CTGCTGCCACCAGCTTAAC |
| Exon1 | TGCTTGGTTTCTGTAATGAGG | GAACAACCCATCTCAAAGCTG |
| Exon2 | CCAGATTCCTCAAGCTGTGTC | TCGTTTACACCCTTTCTCTGC |
| Exon3 | TTTGTGGTTGGCTGACATTCT | TCTACAAGCACTCAAGGTGGG |
| Exon4 | ATCAATAACGTGGGTGTCGTG | CTAGGATCGAAACCCAAGCC |
| Exon5 | GAGGCTCTGAGGATGAGGATT | TGAGCTGTTTCTCTCACCTGG |
| Exon6 | ATTACCTGCTTCTGCTGGACTG | TGCCCAGTGAACACACAATG |
| Exon7-8 | ATTGCCTCTCGATGGTTCAG | CAAGAACATGTCTCTCTGCCC |
| Exon9 | CAGCCATGTAGACAAGGGGT | CAGGAATTCCACAGGCAGGT |
| Exon10 | GTGCCTGGAACATAATGGAGA | ATGATACACTCCCTGGCACAC |
| Exon11 | TTGCAAAGTGTAATGTGCAGG | CACCTTGAGGATGCCTGAGT |
| Exon12 | TGTAATGACGTGACCTCCCTC | CAGCCGAGCAAAGATTCTATC |
| Exon13 | GTGTGAATCACTGTGCCCTG | TGAGGATGTGAGGCTCTCTGT |
| Exon14 | CCCTTGGCAAGTGTGTCTGT | GGTCCTTCTGGTGCTTAAAGG |
| Exon15 | GCTGGAGGGCTAAGTCTCACT | AAGGGAGCCTCCATCTCATAC |
| Exon16 | CATAACTGTCACAGCCACCCT | GTCTCCATCTCCTGATCTCGT |
| Exon17 | CCTTTCCCCTGATTTCCTACC | TCACCTTTTGGGAACAGACAC |
| Exon18 | AGGATTACAGAGGCCACCAGT | GATTTTAGGGCAGTCACACCTC |
| Exon19 | GGGCTCCATAACTAAAGTTTCTTTC | CAGACCACTGACTGCCACCT |
| Exon20 | TTTGAAATGCTCCATCTCCAG | TGATCAGCTTCGTGAGTCCTT |
| Exon21 | ACCCCAGGGAGTCTTTTCTGT | GAGATTCCTGCTTCCTCTGGT |
| Exon22 | ACCTTTCGTCATCTTCATCCA | GAAGGCAATGCTACGTCAGAG |
| Exon23 | CAAGAGTTTGGTGTTTCTGCC | GAGCATATTCGACAGCTTTGC |
| Exon24 | CAAATGGAAACCCCTTTGC | CGTGAACCATCGCACCC |
| Exon25 | GAGCCCTAGGGTTATCAGAGG | GAGGCTTCCATTCTAGGGTGA |
| Exon26_1 | CTCCCATGTCTTCTCCCTG | CTGCTGGTTGATTTCCGATCT |
| Exon26_2 | CAGCATCGTGCTCACCAAAG | CAGTTCCCAGGACCATCACT |
| Exon27_1 | GACCTGGGCCACTTAGTTCA | CCGTTTTAAACTGGTGGGTTT |
| Exon27_2 | CCATTCTGAAGAAAGGAACTGC | CTTAACTGTGGCTGGTGATGG |
| Exon27_3 | AGTTCTTTGGTTGGAAGGAGC | CTTAACTGCCTCAGTTGTGCC |
| Exons exceeding 700pb in length were amplified as multiple sets (eg. Exon 26_1, Exon 26_2).  Exons 7 and 8 were amplified as one unit because of their small size.  Promoter A and B primers sets were used to amplify the promoter region of *INPP5D* | | |

# Supplementary Figures


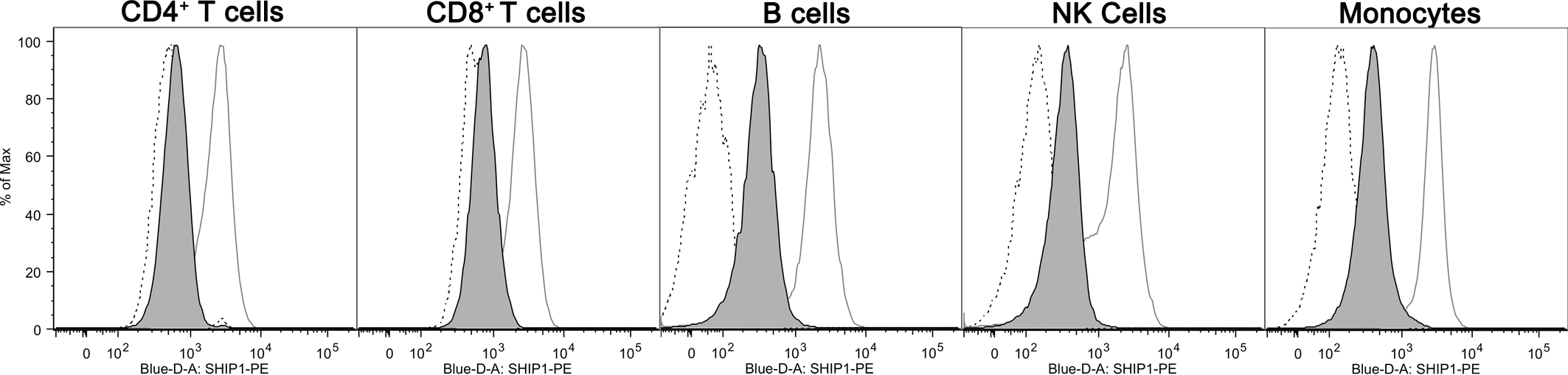


**Supplementary Figure 1. *ATG16L1* Single Nucleotide Polymorphism (SNP) rs2241880 CD-risk allele (GG) does not correlate to reduced SHIP1 mRNA expression or to SHIP1 deficiency**. (A). No significant differences were observed in the level of SHIP1 mRNA expression between the different genotypes of SNP rs2241880 in all subjects. (SHIP1-deficient, red, n=11; SHIP1-sufficient, black, n=61; healthy controls, blue, n=19). No significant differences were observed in the frequency of each genotype between (B) SHIP1-deficient, SHIP1-sufficient, (Fisher’s exact test AA vs GG P=0.6910, GA vs GG P=0.1371, AA or GA vs GG P=0.1627) or (C) in SHIP1-deficient CD vs CD SHIP1-suffucient CD (Fisher’s exact test AA vs GG P=1.000, GA vs GG P=0.3382, AA or GA vs GG P=0.3911).


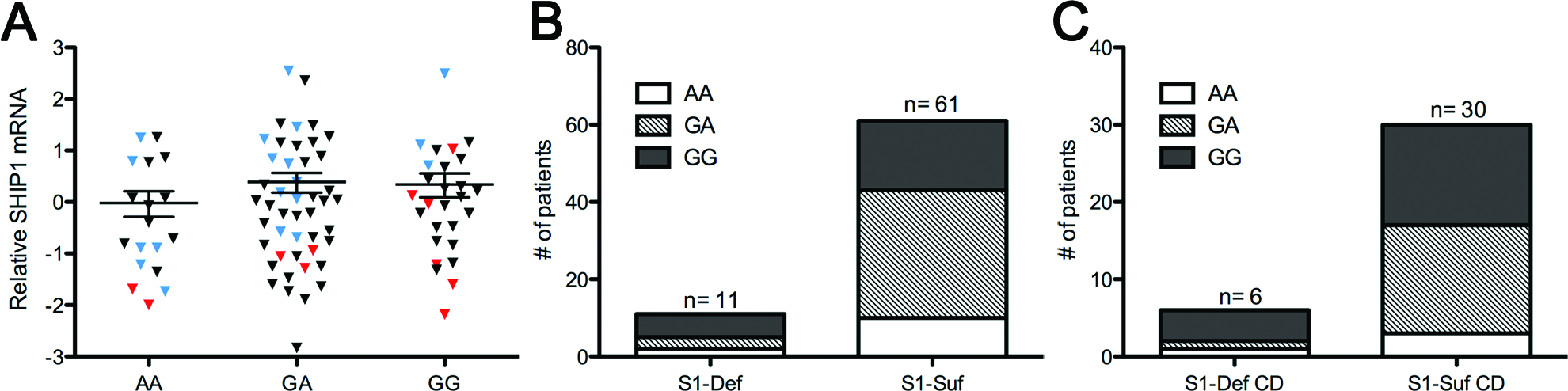


**Supplementary Figure 2. SHIP1 deficiency is observed in all major blood lineages**. (A) Histograms for SHIP1 staining by icFlow performed on WBC from a SHIP1-deficient (BK003, black line-grey fill) and SHIP1-sufficient (BK094, grey line) IBD patient, with FMO (dotted line). Samples were first gated for total live cells, (Fig. 5A) then for T cells (CD3+, CD56-, Fig. 5A), CD4 T cells (CD3+CD4+CD8-), CD8 T cells (CD3+CD4-CD8+), B cells (CD19+ CD3-), NK cells (CD56+CD3-), monocytes (CD14+) or neutrophils (CD15+CD16+, Fig. 5A).

**
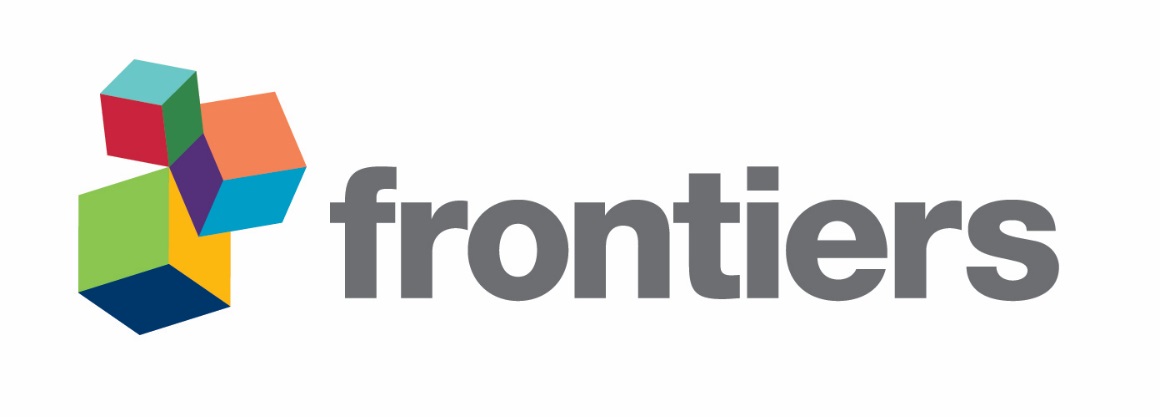
**
